# Gut bacteria induce IgA expression in pituitary hormone-secreting cells during aging

**DOI:** 10.1016/j.isci.2023.107747

**Published:** 2023-08-26

**Authors:** Yehua Li, Jiawen Wang, Rui Wang, Ying Chang, Xiaodong Wang

**Affiliations:** 1 National Institute of Biological Sciences, 7 Science Park Road, Zhongguancun Life Science Park, Beijing 102206, China; 2 Tsinghua Institute of Multidisciplinary Biomedical Research, Tsinghua University, Beijing, China

**Keywords:** Immunology, Microbiome, Endocrinology

## Abstract

Pituitary hormone decline is a hallmark of aging. However, the precise gene regulation mechanism during pituitary aging is unclear. Here, we characterized the cell population alteration and global transcriptional change during pituitary aging through single-cell RNA sequencing (scRNA-seq). We found that mRNA-encoding components of protein translational machinery declined the most in the pituitary during aging. Remarkably, Immunoglobulin A (IgA) was found to be expressed in hormone-secreting cells, and the IgA expression level increased dramatically in aged pituitary. Moreover, the pituitary IgA expression was regulated by gut microbiota. The non-hematopoietic origin of the IgA+ cells in the pituitary was further confirmed through bone marrow transplantation. Somatotropes were identified as the most prominent IgA-producing cells through lineage tracing. Thus, pituitary hormone-secreting cells can generate IgA in an age-dependent manner, and such a process is influenced by gut bacteria.

## Highlights

• Protein translational machinery decline in aged pituitary hormone-secreting cells
• B cell-independent IgA expression increases in aged pituitary
• Gut bacteria regulate IgA expression in pituitary cells
• Somatotropes are the prominent IgA-producing hormone-secreting cells

## Graphical abstract

**Figure:**
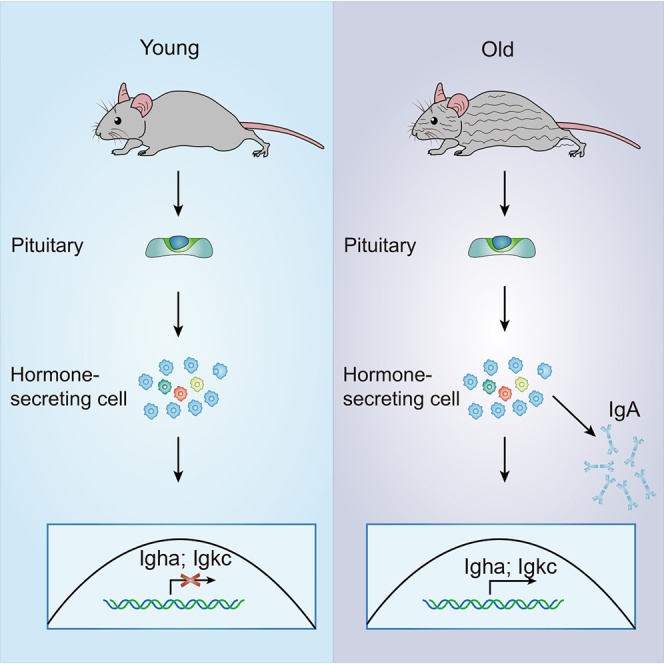

## Introduction

The pituitary gland is the master endocrine organ that drives highly conserved physiologic processes in mammals.^1^^,^^2^^,^^3^ The anterior pituitary is the major hormone secretion gland that consists of five types of endocrine cells: growth hormone (GH) secretion somatotropes, adrenocorticotropin (ACTH) secretion corticotropes, thyroid-stimulating hormone (TSH) secretion thyrotropes, sex hormone secretion gonadotropes, and prolactin (PRL) secretion lactotropes.^4^^,^^5^ Besides hormone-secreting cells, other supporting cells, including mesenchymal cells, endothelial cells, and macrophages are important components of the normal pituitary.^6^^,^^7^

Pituitary hormone alteration, such as GH decline, is a hallmark of mammalian aging.^8^^,^^9^ Although many studies address the cause and effect of pituitary hormone changes during aging, especially the GH-IGF-1 (insulin-like growth factor 1) axis, a global transcriptional picture showing the different types of cell alteration and gene expression changes at individual cell resolution during the pituitary aging process is still missing.

IgA is a unique type of antibody secreted to the mucosal surface, and its dimerization and secretion are facilitated by covalent attachment to the J chain.^10^^,^^11^ Secretory IgA in mucosal surfaces, mainly intestinal epithelium, helps to provide a physical barrier to defend against foreign pathogens, including bacteria and viruses.^12^^,^^13^^,^^14^ In addition, IgA provides exquisite control of the microbiota within the intestine by affecting bacterial colonization and gene expression. Concurrently, microbiome signals also stimulate IgA production.^15^^,^^16^ Besides functions on epithelial surfaces, IgA is also found to regulate neuro-inflammation and defend against infections in the meninges.^17^^,^^18^ IgA has been known to be produced by plasma cells, the terminally differentiated B cells.

In this study, we provided a comprehensive scope of cell populations and gene expression changes during pituitary aging with individual cell resolution. The results showed that translational dysfunction, but not transcriptional decline, might be the cause of hormonal alteration during pituitary aging. Surprisingly, this study also revealed IgA generation in the hormone-secreting cells during aging of the pituitary, and such a process is influenced by the gut microbiome.

## Result

### Messenger RNAs-encoding protein translational machinery declined in pituitary hormone-secreting cells during aging

**Figure 1:**
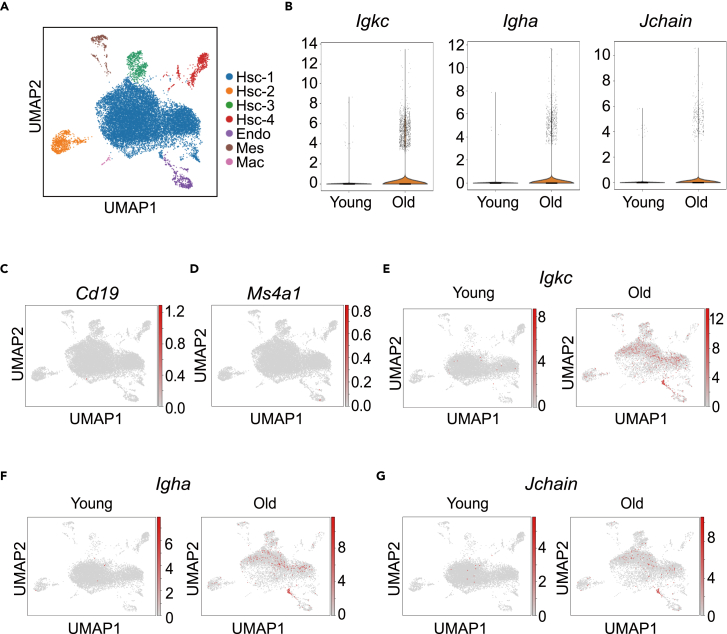
Single-cell RNA-seq (scRNA-seq) analysis reveals IgA+ cells increase in old mouse pituitary (A) ScRNA-seq of the pituitary from young (2–3 months old) and old (18–24 months old) female mice using the 10x Genomics Chromium platform. Uniform manifold approximation and projection (UMAP) clustering of 16,214 single-cell transcriptomes (9,407 from young and 6,807 from old) colored by cell-type clusters. Hsc represents for hormone-secreting cells, Endo for endothelial cells, Mes for mesenchymal cells, and Mac for macrophages. (B) Violin plot showing expression of immunoglobulin kappa constant (*Igkc*), immunoglobulin heavy constant alpha (*Igha*), and *Jchain* as log-normalized counts in young and old pituitary cells. Each dot represents the expression level in one single cell. *Igkc*∗∗∗p = 4.72x10^−28^, *Igha*∗∗∗p = 6.23x10^−8^, *Jchain*∗p = 0.03. p values are false discovery rate (FDR)-corrected, Wilcoxon differential expression test. (C and D) Scatterplots showing the expression of *Cd19* (C) and *Ms4a1* (D) in mice pituitary cells projecting on the UMAP plot. Gray to red coloring indicate low to high-expression levels. (E–G) Scatterplots showing the expression of *Igkc*, *Igha,* and *Jchain* in young and old mice pituitary projecting on the UMAP plot. Gray to red coloring indicate low to high expression levels.

To study cell population alteration and better characterize gene regulation during pituitary aging, we performed single-cell RNA sequencing (scRNA-seq) of cells isolated from the pituitary of young (2–3 months old) and old (18–24 months old) C57/B6 mice. Blood was removed from the mice by perfusion, and pituitary cells were dissociated as previously reported.^19^^,^^20^^,^^21^ Four young or old pituitaries were mixed together and then the live cells were sorted and collected by fluorescence-activated cell sorting (FACS). The scRNA-seq was performed on the sorted cells using the 10x Genomics Chromium platform. The analysis of 16,214 (9,407 from young and 6,807 from old) high-quality single-cell transcriptomes with uniform manifold approximation and projection (UMAP) revealed 7 distinct cell types (Figures 1A and S1A). Characterization of markers in these clusters identified them as several types of hormone-secreting cells (Hsc-1 to 4), endothelial cells (Endo), mesenchymal cells (Mes), and macrophages (Mac) (Figures S1B and S1C). When comparing the cell populations in young and old pituitary, we observed a shift in the largest population of hormone-secreting cells Hsc-1, while the other cell populations remained similar (Figures S2A and S2B).

To define the specific change of Hsc-1 during aging, we compared the gene expression profiles of Hsc-1 in young and old pituitaries. Pathway analysis of differentially expressed genes showed that the genes functioning in ribosome-mediated cytoplasmic translation showed the most drastic decline in expression during aging (Figure S2C). Among the top 10 downregulated pathways in aged Hsc-1, 8 were ribosomal subunits, indicating that protein translation decline in hormone secretion cells was the most significant change during pituitary aging. This phenomenon is consistent with the previous observation that pituitary hormones, products of the protein translational machinery, decrease in aged mice.^8^^,^^9^ Among the top 10 upregulated pathways, 4 were transcription related, which might constitute a compensatory response to the translational decline in Hsc-1 cell populations in the aged pituitary.

### Increase of B cell-independent IgA expression in aged pituitary

While investigating the gene expression change of Hsc-1 cells in aged pituitary, we noticed that *Igha* (IgA heavy chain), *Igkc* (kappa light chain), and *Jchain*, the three genes contributing to the assembly of dimeric IgA, were among the top upregulated genes (Figure S2D). Since Hsc-1 clustered as pituitary hormone-secreting cells, this result was surprising. Consistent with *Igha*, *Igkc*, and *Jchain* increase in Hsc-1, the number of cells expressing these three genes increased dramatically in the aged pituitary. (Figure 1B). The expression of B cell marker genes *Cd19* and *Ms4a1* (Cd20) was mostly absent in the pituitary regardless of age, suggesting the non-B cell origin of the IgA+ cells in the old pituitary (Figures 1C and 1D). Further investigation of single-cell transcriptomic data showed that *Igkc*, *Igha*, and *Jchain* were indeed expressed in some of the hormone-secreting cells (Figures 1E–1G).

**Figure 2:**
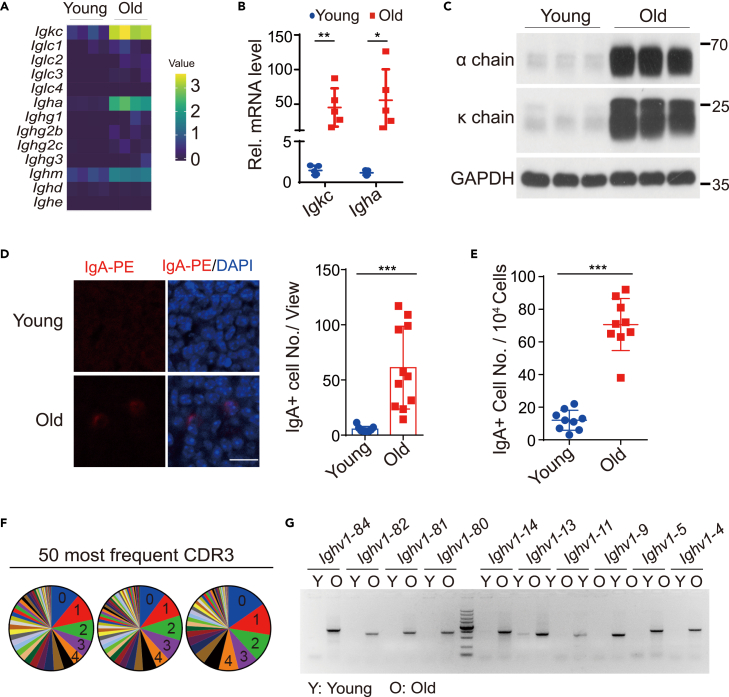
Increase of clonal heterogeneous IgA+ cells in old pituitary (A) Heatmap showing the normalized expression of immunoglobulin light chains and immunoglobulin heavy chains in young and old pituitary. n = 4 samples per group. Gray to red indicate low to high-expression levels. (B and C) Quantitative polymerase chain reaction with reverse transcription (qRT-PCR) (B) and Immunoblotting (C) showing mRNA and protein expression of *Igkc* (κ chain) and *Igha* (α chain) in young and old pituitary. n = 4 mice for young and n = 5 mice for old in B. Representative data of three independent experiments for immunoblotting is shown in C. (D) Immunohistochemistry image (left) and quantification (right) of IgA+ cells in the pituitary of young (n = 9) and old (n = 11) mice. Paraffin sections of pituitary from young and old mice were stained with 4′,6-diamidino-2-phenylindole (DAPI) and IgA-PE antibody as described in STAR Methods. Blue is DAPI signal and red is IgA signal. Data were compiled from three independent experiments. Scale bar, 20μM. (E) Quantitation of the IgA+ cells in young and old pituitary. Pituitary cells from young and old mice are stained with IgA-PE antibody and analyzed by Fluorescence-activated cell sorting (FACS) as described in STAR Methods. n = 9 mice per group. (F) Pie charts depicting the relative abundance of 50 most frequent complementarity-determining region 3 (CDR3) amino acid sequences of immunoglobulin α heavy chain in aged pituitary from three mice. Data shown as mean ± s.d., unpaired two-tailed Student’s *t* test. ∗p < 0.05, ∗∗p < 0.01, ∗∗∗p < 0.001. (G) PCR result showing selected v chains of IgA heavy chain expression in young and old pituitary. Primers specific for *Igha* are used for reverse transcription of mRNA from young and old mice pituitary and selected v chains are amplified by PCR. The darkest DNA marker band is 1000bp. Image is representative data of three independent experiments.

To confirm that it was an age-dependent generation of IgA, we did transcription profiling of young and old pituitary. Circulating immune cells in blood were removed from the mice by perfusion, and mRNA was extracted from the whole pituitary for next-generation sequencing. The *Igkc* and *Igha* mRNA levels were significantly increased in the old pituitary, while expressions of genes encoding other antibody subtypes were largely unchanged (Figure 2A). Additionally, qRT-PCR and *in situ* hybridization results further confirmed the upregulation of *Igkc* and *Igha* mRNA in old pituitary (Figures 2B and S3A). At the protein level, immunoblotting of perfused pituitary cell lysates showed a pronounced increase of immunoglobulin κ chain and α chain, products of *Igkc* and *Igha*, in old pituitary (Figure 2C). The immunohistochemistry (IHC) staining using an antibody against IgA heavy chain also showed positive signals in the pituitaries of old mice (Figure 2D). To better quantify the IgA+ cells, pituitaries from young or old mice were dissociated to single cells and stained with Phycoerythrin (PE)-conjugated anti-IgA antibody after fixation. The samples were then subjected to FACS analysis to calculate the percentage of IgA+ cells in the whole pituitary. The percentage of IgA+ in old pituitary increased significantly, although the overall percentage was low (Figures 2E and S3B).

In addition to the pituitary, we also checked the mRNA expression of *Igha* in other organs. Compared to young animals, the *Igha* mRNA level also increased serval fold in the liver and kidney of old mice, but the changes were much lower than that in the pituitary (>30-fold). The *Igha* mRNA levels deceased in old spleen and were unchanged in the intestine, lung, and brain (Figure S3C). These results indicated that IgA elevation in the pituitary was not a result of global immune system change during aging.

### Clonal heterogeneity of the IgA+ cells in old pituitary

Antibody specificity is determined by its variable region (V-region). The V-region-encoding genes are primarily assembled from the germline V, D, and J gene segments by V(D)J recombination. To check the clonal characteristics of IgA+ cells in old pituitary, we analyzed the RNA-seq data and found the repertoire of V segments utilized in immunoglobulin heavy chain and light chain is comparable between the young and old pituitaries, indicating a preselected nature of the pituitary IgA (Table S1). To further analyze the IgA repertoire in the old pituitary, the RNA was isolated from perfused pituitary and transcribed into cDNA with primers specific for the constant region of *Igha* (*Ca*), and BCR sequences were amplified by PCR with primers annealing to FR1 (framework region 1) and the Ca domain. The amplicons were sequenced, and CDR3 diversity was analyzed. Among the top 50 CDR3 of each sample, the frequency of the top 5 was at an equal level, and there was no one dominant CDR3 sequence (Figure 2F). We also used paired primers specifically targeting different V chains and Ca to amplify BCR with different V chains. Consistently, both the distal and proximal V chains in the genome are expressed higher in the old pituitary (Figure 2G). Those data collectively suggested the clonal heterogeneity characteristics of IgA+ cells in aged pituitary.

### Pituitary IgA level is positively correlated with gut-bacteria diversity

**Figure 3:**
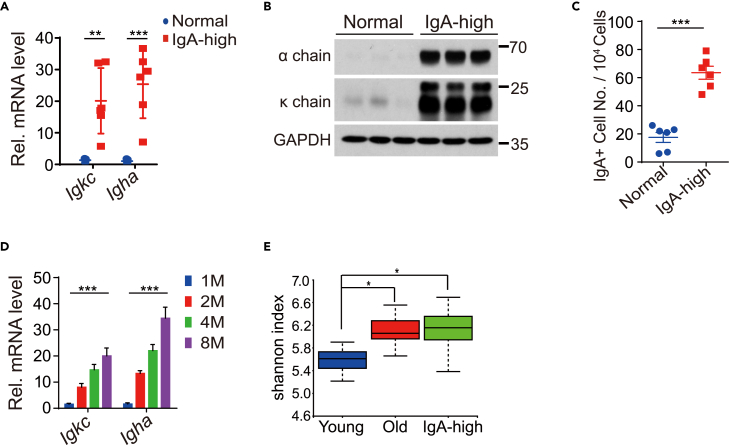
IgA-high mice are with higher gut-bacteria diversity (A and B) qRT-PCR (A) and immunoblotting (B) showing mRNA and protein level of *Igkc* (κ chain) and *Igha* (α chain) in normal and IgA-high mouse pituitary. n = 5 mice for Con and n = 6 mice for IgA-high in A. Representative data of three independent experiments is shown in B. (C) FACS analysis and quantitation of IgA+ cells in pituitary of normal and IgA-high mice. n = 6 mice per group. (D) *Igkc* and *Igha* mRNA level in pituitary of IgA-high mice of different ages. M as Month, n = 6 mice for 1M, 2 M, 4M and n = 5 for 8M. (E) 16S rRNA gene profiling data comparing the gut microbiome of young, old and IgA-high mice. Alpha diversity indicated by Shannon index is shown. n = 9 mice in young and old, n = 10 mice in IgA-high. Data shown as mean ± s.d., unpaired two-tailed Student’s *t* test for a and c, one-way ANOVA test for d and Kruskal Wallis test for e. ∗p < 0.05, ∗∗p < 0.01, ∗∗∗p < 0.001.

In addition to age-related IgA elevation, we also found that pituitary IgA levels varied among different mice, with one particular group expressing the highest level at 3 months old was thus labeled as IgA-high mouse (IgA-high). The mRNA levels of *Igkc* and *Igha* in the pituitary of IgA-high mice were higher than in other mice (Figure 3A). Immunoglobulin κ chain and α chain proteins measured by Western blotting also increased markedly in the 3-month-old IgA-high mice (Figure 3B). To quantify the IgA+ cells in the IgA-high group, pituitary cells from the 3-month-old normal and IgA-high mice were stained with PE-conjugated anti-IgA antibody and analyzed by FACS. As shown in Figures 3C and S4A, the IgA+ cell population increased about 3-fold in IgA-high mice. Similar to the age dependency of normal mice, the gene expression level of *Igha* and *Igkc* in the IgA-high mice also increased as the mice grew older (Figure 3D).

**Figure 4:**
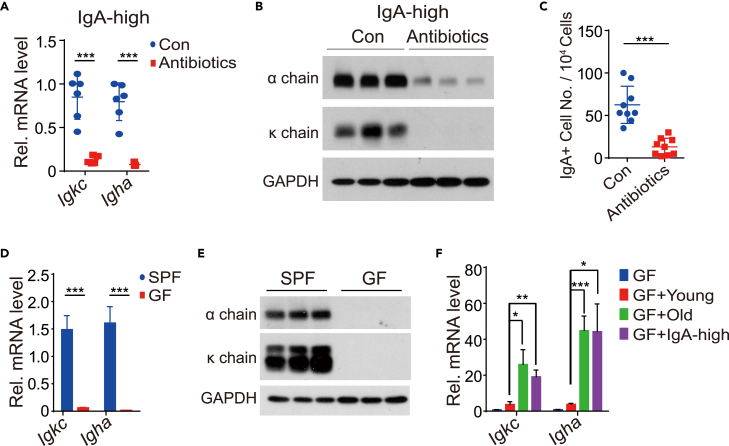
Microbiota determines the amount of IgA+ cells in pituitary (A and B) mRNA (A) and protein (B) level of *Igkc* (κ chain) and *Igha* (α chain) in IgA-high mouse pituitary without (Con) or with (Antibiotics) antibiotics treatment. n = 6 mice for Con and n = 5 mice for Antibiotics in A. Representative data of three independent experiments is shown in B. (C) FACS analysis and quantitation of IgA+ cells in pituitary of control and antibiotics treated mice. n = 9 mice per group. (D and E) qRT-PCR (D) and Immunoblotting (E) result showing *Igkc* (κ chain) and *Igha* (α chain) expression in pituitary of specific pathogen-free (SPF) and germ-free (GF) mice. n = 5 mice per group in D. Representative data of three independent experiments is shown in E. (F) qRT-PCR result showing mRNA level of *Igkc* and *Igha* in GF mice and GF mice transferred with microbiota from young, old and IgA-high mice as indicated. n = 6 mice per group. Data shown as mean ± s.d., unpaired two-tailed Student’s *t* test. ∗p < 0.05, ∗∗p < 0.01, ∗∗∗p < 0.001.

Since IgA-high mice were not genetically different from normal mice and IgA expression was reported to be affected by gut microbiota,^15^^,^^22^^,^^23^^,^^24^ we characterized the gut-bacterial microbiome through 16S rRNA sequencing in young IgA-high mice compared with that of normal mice in both young and old age. The ileocecal microbiome was chosen as it was known to encompass much of the diversity within the gastrointestinal tract.^25^ The microbiome community structure of normal young and old mice or IgA-high young mice was assessed using principal coordinate analysis (PCoA) based on unweighted and weighted UniFrac distances. As shown in Figures S4B and S4C, the gut microbiome of normal young mice clustered together, while that of the normal old mice and IgA-high young mice overlapped. This result indicated the similarity of gut microbiota of normal old mice and IgA-high young mice, which differed from that of normal young mice. Further analysis showed bacterial diversity was lower in normal young mice compared with either normal old or IgA-high young mice (Figure 3E). To test whether gut-bacteria diversity controlled the expression of IgA in the pituitary, we orally treated IgA-high young mice with antibiotics for 2 months to reduce the gut-bacteria diversity (Figure S4D). As shown in Figures 4A and 4B, antibiotics treatment inhibited mRNA and protein expression of *Igkc* and *Igha* in the pituitary of those mice. FACS analysis of IgA+ cells in the pituitary of antibiotics treated group showed that IgA+ cells decreased to one-fifth of the untreated group (Figure 4C). These results confirmed that the pituitary IgA level was regulated by the diversity of gut bacteria.

To better characterize the relationship between gut bacteria and IgA expression in the pituitary, we transferred microbiomes from the normal young and old as well as IgA-high young mice to the young germ-free (GF) mice. The *Igkc* and *Igha* expression in GF mice pituitary were negligible compared with the specific pathogen-free (SPF) mice (Figures 4D and 4E). However, after we transferred the gut microbiome from different mice to the GF mice, the *Igkc* and *Igha* expressions were restored. Furthermore, the GF mice receiving microbiome from the normal old or IgA-high young mice expressed higher levels of IgA in their pituitaries than those that received microbiome from normal young mice (Figure 4F). Collectively, our results indicated that the diversity of gut bacteria controlled the IgA expression in the pituitary.

### The hormone-secreting cells that express IgA

**Figure 5:**
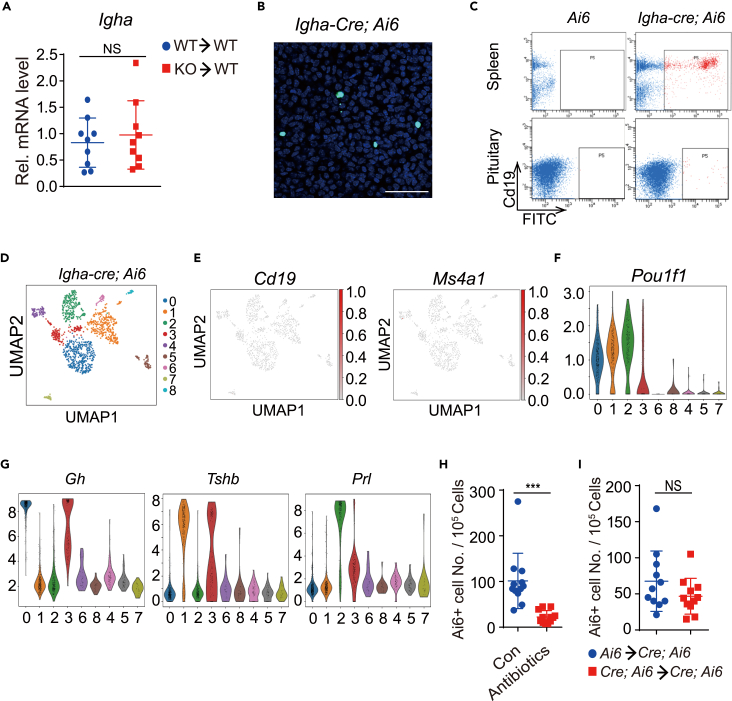
Different types of pituitary hormone-secreting cells express IgA (A) *Igha* mRNA level in pituitary of IgA-high mice transferred with bone marrow from wildtype (WT) and IgA knockout (KO) mice. The mice were sacrificed 4 weeks after bone marrow transfer and pituitary mRNA was analyzed by qRT-PCR. n = 9 mice per group. Data shown as mean ± s.d., unpaired two-tailed Student’s *t* test. NS p > 0.05. (B) Immunohistochemistry image of pituitary from *Igha-cre*; *Ai6* mouse. Green signal indicates ZsGreen expression and DAPI stains cell nucleus is blue. scale bar, 50μM. (C) Analysis of spleen and pituitary cells from *Ai6* and *Igha-cre*; *Ai6* mice. Spleen and pituitary cells from different mice are dissociated and stained with CD19 antibody and analyzed by FACS. Representative data of three independent experiments is shown here. (D) ScRNA-seq of the FACS sorted ZsGreen-positive pituitary cells from *Igha-Cre; Ai6* female mice (4–8 months old) using the 10x Genomics Chromium platform. UMAP clustering of 1,339 single-cell transcriptomes colored by cell-type clusters is depicted. (E) Scatterplots showing the expression of *Cd19* and *Ms4a1* (*Cd20*) in ZsGreen positive pituitary cells from *Igha-Cre; Ai6* mice projecting on the UMAP plot. Gray to red indicate low to high-expression levels. (F and G) Violin plot showing expression of *Pou1f1*, *Gh*, *Tshb*, and *Prl* as log-normalized counts in ZsGreen-positive pituitary cells from *Igha-Cre*; *Ai6* mice. Each dot represents expression level in one single cell. (H) FACS analysis and quantitation of ZsGreen-positive pituitary cells from *Igha-cre*; *Ai6* mice in control or antibiotics treated groups. n = 12 mice per group. (I) FACS analysis and quantitation of ZsGreen-positive pituitary cells of *Igha-cre*; *Ai6* mice transferred with bone marrow from *Ai6* or *Igha-cre*; *Ai6* mice. n = 11 mice per group. Data shown as mean ± s.d., unpaired two-tailed Student’s *t* test. NS p > 0.05, ∗∗∗p < 0.001.

Our scRNA-Seq data indicated that the IgA+ cells in the pituitary were from hormone-secreting cells but not conventional B cells. To validate this finding and rule out the possibility that those IgA+ cells were the result of B cell contamination, we conducted a bone marrow transfer experiment using IgA knockout mice with *Igha* constant region exon2 deleted as doner (Figure S5A). The recipients were IgA-high young mice irradiated with a lethal dose of gamma rays and transferred with the bone marrow cells from wild-type or IgA knockout mice. Four weeks after transfer, the mRNA level of *Igha* in the recipient mice spleens and pituitaries was measured by qRT-PCR. The spleen *Igha* mRNA level in *Igha* knockout bone marrow recipients was much lower than that received wild-type bone marrow cells (Figure S5B). However, no difference in *Igha* mRNA levels was observed between mice that received wild-type or *Igha* knockout bone marrow cells, and the levels were comparable to that of non-irradiated mice (Figure 5A), indicating that IgA was generated in non-hematopoietic cells.

We also sorted Epcam+ hormone-secreting cells of normal young and old mice pituitaries to further examine the *Igkc* and *Igha* expression. The Epcam+ cells in the old pituitary expressed significantly higher levels of *Igkc* and *Igha* than the cells from the young pituitary (Figure S5C). These data further supported the notion that IgA in the old pituitary was generated by hormone-secreting cells rather than circulating B cells.

Since the finding that IgA was produced in the hormone-secreting cells from the pituitary was highly surprising, we tried to further verify this finding by generating *Igha-cre* mice. After crossing *Igha-Cre* mice with the *Ai6* reporter mice, all cells that accomplished V(D)J recombination and class-switching to IgA would drive Cre expression and delete the stop codon before *ZsGreen* gene to induce its expression. Therefore, the cells that had expressed IgA would be labeled by ZsGreen. To obtain ZsGreen signal in the mouse pituitary, *Igha-cre; Ai6* male mice were crossed with IgA-high female mice, and the male mice were removed one week later. This way, the maternal microbiota from IgA-high mice would be transferred to the offspring naturally and make the *Igha-cre; Ai6* mice with higher IgA expression.^26^^,^^27^ In the intestine of those mice, ZsGreen signal was specifically detected in payer’s patch and lamina propria, places where IgA plasma cells reside (Figure S5D). In the spleen, most ZsGreen positive cells were also positive for B cell marker *Cd19* (Figure 5C), indicating successful *Igha*-dependent generation of ZsGreen signal. The ZsGreen signal was detected in the pituitary of those mice as well (Figure 5B). However, the ZsGreen-expressing cells in the pituitary were CD19 negative, a feature that was different from that in the spleen (Figure 5C). ZsGreen-positive cells from the pituitaries of *Igha-Cre*; *Ai6* mice were then sorted and subjected to scRNA-seq. We obtained 1,339 single-cell transcriptomes and divided them into 9 distinct cell types by UMAP analysis (Figure 5D). Marker genes identified them as different types of hormone-secreting cells (cluster 0, 1, 2, 3, 6, and 8), macrophages (cluster 4), mesenchymal cells (cluster 5), and a unique group that expressed the highest level of IgA (cluster 7) (Figure S5E). B cell markers *Cd19* and *Ms4a1* were not detected in most *Igha-Cre*-traced pituitary cells (Figure 5E). Different from cluster 7 in which *Igha* and *Igkc* were highly expressed, other cell clusters express IgA at low levels (Figure S5F). Further analysis of the *Igha-Cre* traced hormone-secreting cells revealed most cells (cluster 0, 1, 2, and 3) were *Pou1f1* positive (Figure 5F). The top 3 clusters (cluster 0–2) with the largest number of cells were somatotropes (cluster 0, 30%), thyrotropes (cluster 1, 24%), and lactotropes (cluster 2, 18%), respectively (Figure 5G). Consistently, IgA expression could be observed in *Pit1-Cre* traced hormone-producing cells from the pituitary of 20M old mice (Figure S5G). These data collectively supported that different hormone-secreting cells in pituitary expressed IgA and somatotropes were the most prominent IgA-producing cells.

Using those *Igha-Cre*; *Ai6* mice, we further validated the relationship between gut-bacteria diversity and IgA+ cells in the pituitary. The *Igha-Cre*; *Ai6* mice were treated with antibiotics to reduce gut-bacteria diversity, and ZsGreen-positive cells in the pituitary were quantified. Consistently, ZsGreen-positive cells in pituitary decreased dramatically after antibiotics treatment (Figure 5H). We also tested the contribution of bone marrow cells to ZsGreen-positive cells in the pituitary. *Igha-Cre*; *Ai6* reporter mice were irradiated with a lethal dose of gamma ray and transplanted with bone marrow cells from *Ai6* or *Igha-Cre*; *Ai6* mice. The ZsGreen-positive cells in pituitaries were quantified one month later. The FACS data showed that the ZsGreen-positive cells in recipient mice were at equal levels regardless of the genotype of the donor (Figure 5I). Thus, IgA in the pituitary was again confirmed to originate from non-hematopoietic cells.

### IgA in pituitary prevents virus infection

Lastly, we tried to figure out the function of IgA in the pituitary. Previous report shows IgA plays an important role in trapping microbes and protecting the body from pathogens challenge.^14^^,^^28^^,^^29^ Given the pituitary is closely related to the circulation system, it may easily be attacked by pathogens in blood and IgA may play some protective roles. To test this hypothesis, we infected the 3–4 months old control and IgA-high mice with Adeno-associated viruses (AAV) expressing EGFP by tail vein injection. Two weeks after virus infection, EGFP expression could be detected in the pituitary, indicating the pituitary could be successfully infected by AAV. Then we compared the infection efficiency in control and IgA-high mice after AAV injection by quantitating EGFP-positive cells in the pituitary. The results showed less EGFP-expressing cells in IgA-high mice pituitary, indicating IgA expressed in pituitary could protect it from virus infection (Figures S6A and S6B). Since the global diminished function of immune system in old mice would promote virus infection, we did not compare the AAV infection in young and old pituitary. The functional test suggested IgA in the pituitary prevents it from virus attack, and it might be a compensation for immune aging.

## Discussion

The current study revealed that decreasing mRNAs-encoding protein translational machinery was one of the most significant changes in aged pituitary hormone-secreting cells. This phenomenon could explain the pituitary hormone decline during aging. Consistent with our data, a recent study reported that increased ribosome pausing contributes to proteostasis impairment during aging.^30^

Surprisingly, scRNA study of young and old mouse pituitary revealed IgA expression in the hormone-secreting cells in aged pituitary. This finding is remarkable since plasma cells are the only known source of IgA antibodies.^31^^,^^32^ Although immunoglobulin genes were reported to express in non-B cells previously, the data did not convincingly exclude B cell contamination, and the conclusion had been challenged.^33^^,^^34^^,^^35^ We therefore tried several experimental approaches aiming to eliminate the possibility of B cell contamination in the old pituitaries: (1) identification of *Igha*, *Igkc*, and *Jchain* expression by single-cell sequencing in a B cell-free condition; (2) bone marrow transplant from IgA knockout mice; (3) scRNA sequencing of *Igha-cre*; *Ai6* traced IgA-producing cells from pituitaries further validated their identities as hormone-secreting cells and revealed somatotropes were the most prominent IgA-producing cells.

The finding of gut-bacteria diversity regulated IgA production in the pituitary was consistent with previous findings that gut bacteria influence IgA plasma cells in the meninges.^18^ However, the meninges-residing IgA plasma cells were proposed to be educated in the gut and circulated to the meninges after maturation, which was different from our observations that circulating B cells did not affect IgA expression in aged pituitary. It remains unclear how the signals transmitted from gut-bacteria affect IgA gene expression in the pituitary. One possibility may be a specific or combination of metabolites generated by gut bacteria that could be sensed by pituitary cells. How those metabolites are sensed and initiate the activation of IgA gene recombination and expression will be an interesting future research direction. Alternative hypotheses, such as communication of gut bacteria on the nerve of intestines somehow affecting the pituitary, also exist. Nevertheless, the generation of IgA signal in pituitary could be used as a reporting system to study gut-pituitary communication in the future.

Since IgA expressed in pituitary is not at a high level, we are curious about the reason why it is essential, especially during aging. The virus-defending effect of IgA in the pituitary may give us some indication in explaining this puzzle. The central nervous system (CNS) is protected by the blood-brain barrier (BBB).^36^^,^^37^ However, the pituitary gland is directly connected to the hypothalamus through a stalk of blood vessels and nerves (the pituitary stalk), where there is a BBB leakage. Therefore, pathogenic viruses and bacteria in the circulation system may attack the CNS through this hypothalamus-pituitary interaction site. IgA expressed in the pituitary may prevent the pathogens from infecting the CNS system through this hypothalamus-pituitary connection. The protecting role of the IgA in the pituitary may become more critical in old organisms, in which gut bacteria diversity increases while humoral responses decline.

### Limitations of the study

The IgA signal in pituitary cells was very low and could only be detected through scRNA-seq, which impeded further understanding of this issue. We failed to find out the precise regulatory signal of IgA expression in pituitary cells and could not amplify the IgA signal to better confirm and study IgA generation in pituitary cells.

## STAR★Methods

### Key resources table

**Table:** 

REAGENT or RESOURCE	SOURCE	IDENTIFIER
**Antibodies**		

Goat Anti-Mouse IgA alpha chain (HRP)	abcam	ab97235
Goat Anti-Mouse Kappa-HRP	Southernbiotech	1050-05
Anti-GAPDH mAb-HRP-DirecT	MBL	M171-7
goat anti-mouse IgA-PE	Southernbiotech	1040-09
APC anti-mouse CD19 Antibody	Biolegend	115512
FITC conjugated Epcam antibody	Invitrogen	11-5791-82

**Bacterial and virus strains**		

AAV-GFP	Genetic Screening Center of NIBS	N/A

**Chemicals, peptides, and recombinant proteins**		

DAPI (4',6-diamidino-2-phenylindole)	Thermo Fisher Scientific	D1306
ampicillin	Sigma	A9393
vancomycin	Sigma	V2002
metronidazole	Sigma	M1547
neomycin	Sigma	N6386
Amphotericin B	Sigma	A4888
Red Blood Cell Lysis Buffer	Beyotime	C3702-120ml
SuperBlock™ (PBS) Blocking Buffer	Thermo Fisher Scientific	37515
DAPI Fluoromount-G	SouthernBiotech	0100-20
Paraformaldehyde (PFA)	Solarbio	P1110
Probes for mouse Igkc	Advanced Cell Diagnostics	414291
Probes for mouse Igha	Advanced Cell Diagnostics	414281-C2
Opal 520 reagents	Akoya Biosciences	FP1487001KT

**Critical commercial assays**		

Multiplex Fluorescence v.2 kit	Advanced Cell Diagnostics	323100
RT-PCR Kit	Takara	RR014A

**Deposited data**		

Young and old mouse pituitary RNA-seq	This paper	GSE215110
Young and old mouse pituitary single cell RNA-seq	This paper	GSE239390
Igha-Cre; Ai6+ pituitary cells single cell RNA-seq	This paper	GSE239390

**Experimental models: Organisms/strains**		

Mouse: C57BL/6N	Charles River	213
Mouse: Igha-Cre	This paper	N/A
Mouse: Igha KO	This paper	N/A
Mouse: B6 Cg-Gt (ROSA)26Sortm6(CAG-ZsGreen1) Hze/J	The Jackson Laboratory	007906
Mouse: Pit1-Cre	This paper	N/A

**Oligonucleotides**		

*Igha* KO-F: GAAGGTGTTCACTGGAAGGTAG	This paper	N/A
*Igha* KO-R: GGATGTGAAGTGCTCTGGTAAA	This paper	N/A
Cre-F: TTGCCAGGATCAGGGTTAAAG	This paper	N/A
Cre-R: CTTGCATGATCTCCGGTATTGA	This paper	N/A
*Igha*-WT-F: GTCAAGAGCCACTTTCCTATGT	This paper	N/A
*Igha*-WT-R: CCTGGAGTTGGGTTATGTCTTC	This paper	N/A
IgA RT-1: ATCAGGCAGCCGATTATCAC	This paper	N/A
IgA RT-2: TCTCCTTCTGGGCACTCG	This paper	N/A
IgA RT-3: TGAATGATGCGCCACTGT	This paper	N/A
Ca: GAGCTCGTGGGAGTGTCAGTG	This paper	N/A
Vh: AGGTGCAGCTGCAGGAGTCTGG	This paper	N/A
IgA-1: ATCAGGCAGCCGATTATCAC	This paper	N/A
IgA-2: TCTCCTTCTGGGCACTCG	This paper	N/A
IgA-3: TGAATGATGCGCCACTGT	This paper	N/A
PCR primer for V chains, see Table S2	This paper	N/A
Igkc-F: GTTGACCAAGGACGAGTATGAA	This paper	N/A
Igkc-R: TTGAAGCTCTTGACAATGGG	This paper	N/A
Igha-F: CCAGAAGGAGAATCCGTGAAA	This paper	N/A
Igha-R: AGGAGGACAAGGAGGACAA	This paper	N/A

**Software and algorithms**		

GraphPad Prism 9	GraphPad	https://www.graphpad.com/
Lasergene	DNASTAR	https://www.dnastar.com/software/lasergene/
Nikon A1-R	Nikon	https://www.microscope.healthcare.nikon.com/products/confocal-microscopes/a1hd25-a1rhd25
Flowjo10	BD Biosciences	https://www.flowjo.com/

### Resource availability

#### Lead contact

Inquiries and requests for resources should be directed to and will be fulfilled by the lead contact, Dr. Yehua Li (liyehua@nibs.ac.cn).

#### Materials availability

All mouse lines generated in this study will be made available on request to the lead contact; however, requestor will cover shipping costs. This study did not generate new unique reagents.

### Experimental model and study participant details

#### Mice

Mice were bred and maintained in the National Institute of Biological Sciences (NIBS) specific-pathogen-free facility in accordance with the Guide for the Care and Use of Laboratory Animals of NIBS. Procedures were approved by the Laboratory Animal Management Committee of NIBS and were in compliance with all relevant ethical regulations. All specific-pathogen-free mice used in experiments were socially housed under a 12h light–dark cycle with free access to food and water, with 23°C–25°C and 50%–56% humidity. Germ-free mice were maintained and microbiota transfer experiments were done in the facility of Institute of Laboratory Animal Science (CAMS & PUMC). C57BL/6 mice were purchased from Beijing Vital River Laboratory. *Ai6* mice (Jackson stock no. 007906) were provided by Ting Chen. *Igha KO* mice and *Igha-Cre* mice were generated using the CRISPR-Cas9 system. For *Igha* KO mice generation, two gRNAs 5’-tagttgagcatctgtattat-3’ and 5’-cctctgaggcacaccctcac-3’ were used to delete the exon2 of *Igha* constant region. For *Igha-Cre* mice, *P2A* tag and *Cre* was added to the C terminal of *Igha* gene through homologous recombination. The gRNAs used for *Igha-Cre* mice generation were 5’-gggccacaggctggcacctg-3’ and 5’-ggtgaggaaggtcacagtggt-3’. For *Pit1-Cre* (*Pou1f1-Cre*) mice, *P2A* tag and *Cre* was added to the C terminal of *Pou1f1* gene through homologous recombination. The gRNAs used for *Pit1-Cre* mice generation were 5’- ggcgtgtgatagccatgtg-3’ and 5’- ggtgttgtgagggactttcc-3’. The genotyping primer for *Igha KO* mice were *Igha* KO-F: 5’-GAAGGTGTTCACTGGAAGGTAG-3’; and *Igha* KO-R: 5’-GGATGTGAAGTGCTCTGGTAAA-3’, the wildtype allele PCR product is 905 bp and KO product is 220 bp. The genotyping primer for *Igha-Cre* and *Pit1-Cre* mice were: Cre-F: 5’-TTGCCAGGATCAGGGTTAAAG-3’; Cre-R: 5’-CTTGCATGATCTCCGGTATTGA-3’; *Igha*-WT-F: 5’-GTCAAGAGCCACTTTCCTATGT-3’; *Igha*-WT-R: 5’-CCTGGAGTTGGGTTATGTCTTC-3’. The PCR product of Cre is 405 bp and PCR product of *Igha*-WT is 387 bp. *Igha-cre* mouse is Cre positive and there is no band of *Igha*-WT band in *Igha-Cre* homozygous mouse.

### Method details

#### Pituitary gland dispersion

The pituitary glands of mice were dissected, and the tissue was dispersed as previously described.^19^^,^^20^^,^^21^ Briefly, pentobarbital (50mg/kg, i.p.) anesthetized mice were perfused via the aorta with cold PBS and dissected pituitary was placed into an enzyme mix containing 0.5% w/v collagenase type 2 (Worthington, LS004176), 0.25% trypsin (Thermo Fisher Scientific, 25200056), in Hanks balanced salt solution (Gibco, 14175095) for 2-3 hours at 37°C. The tissue and cells were gently triturated and pelleted at 900g for 3 minutes, and resuspended for further single cell sorting. For the young and old pituitary sample, all live cells from young (2-3M) or old (18-24M) pituitary negative for DAPI (Thermo Fisher Scientific, D1306) staining were sorted for further scRNA-seq. For the Epcam+ cells sample, old (18-24M) pituitary cells were stained with DAPI and FITC conjugated Epcam antibody (Invitrogen, 11-5791-82), then DAPI negative and Epcam positive cells were sorted for further processing. For the Igha-cre; Ai6 sample, zsGreen positive and DAPI negative pituitary cells were sorted for sequencing.

#### Single cell library preparation

Single-cell RNA library construction and sequencing Single-cell cDNA libraries were prepared using the Chromium Single Cell 3′ Library and Gel Bead Kit v2 according to the manufacturer’s instructions. In brief, cell suspensions in a chip were loaded on a Chromium Controller (10x Genomics) to generate single-cell GEMs (gel beads in emulsion). scRNA-seq libraries were then prepared using the Chromium Single Cell 3′Gel Bead and Library Kit (P/N 120236, 120237, 120262; 10x Genomics). Qualitative analysis of each DNA library was performed using an Agilent 2100 Bioanalyzer. The concentration of each DNA library was measured using a Qubit fluorometer (Invitrogen). Libraries were sequenced on an Illumina NextSeq 500 (2×150 paired-end reads).

#### Single-cell RNA sequencing (scRNA-seq) analysis

For young and old pituitary, the 10x Cell Ranger (V3.0.2, 10x Genomics) software was used to demultiplex the sequencing data, to map reads to the mus musculus genome assembly mm10 (version 3.0.0, 10x Genomics), to generate feature-barcode matrices and quality control metrics. Then all the feature-barcode matrices were imported into SoupX (v1.6.2)^38^ to removes ambient RNA contamination and then were imported into Scanpy (v1.7.1)^39^ to generate AnnData objects. Cells with detected genes < 500 or >4,000 were filtered. Cells with UMI counts > 40,000 or mitochondrial UMI ratio > 10% were also excluded. Cell doublets were removed from the objects using Scrublet (version 0.2.1)^40^ with the doublet score threshold=0.32. Genes expressed in less than 7 cells were filtered. The objects were concatenated into a single object and genes expression were normalized to 10,000 reads per cell and logarithmized. Then highly variable genes were selected with max_mean=5 and min_disp=0.6. Effects of total counts per cell and the percentage of mitochondrial genes expressed were regressed out using pp.regress_out() function and scaled. Dimensionality reduction was performed by running principal component analysis and then a nearest-neighbor graph was calculated using ‘pp.neighbors()’, followed by clustering using leiden algorithm ‘tl.leiden()’ with a resolution adopting from prior knowledge on pituitary cells population composition. Then uniform manifold approximation and projection (UMAP) dimension reduction was performed for visualizing high-dimensional data. Scanpy rank_genes_groups() and Wilcoxon rank-sum test were used to compute the statistical significance of differential genes expression between any groups and identify marker genes of each cluster. For *Igha-cre; Ai6* sample, ambient RNA contamination removal with SoupX was not applied, and cells with detected genes < 200 or >8,000 were filtered. Cells with UMI counts > 80,000 or mitochondrial UMI ratio > 10% were also excluded. The top 3,000 highly variable genes were selected.

#### Gene set enrichment analysis

Gene Set Enrichment Analysis (GSEA)^41^ was conducted using GSEA software (Version 4.1.0) to identify significantly regulated pathways. The significantly differentially expressed genes (p-value <0.1) were ranked by their log-transformed fold change value. The pre-ranked gene list and MousePath_All_gmt-Format.gmt (http://ge-lab.org/gskb/) were used for running the tool ‘‘Run GSEA Preranked’’ in the classic mode.

#### RNA-sequencing and data processing

The isolated pituitary from young (2-3M) or old (18-24M) mouse was directly lysate in lysis buffer and RNA was extracted using RNAprep pure Cell/Bacteria Kit (TIANGEN, DP430) following the manufacturer’s instructions. Concentration of RNA was measured and the sample passed quality checks was used for RNA sequencing library preparation at NIBS Center for Nucleic Acid Sequencing following manufacturer’s instruction (Illumina). HiSeq 2500 was used for the library sequencing. Fastq files for the sequencing data was assessed by FastQC (v0.11.8) to ensure the good quality and then were mapped with STAR aligner (version 2.6.1a) to the GRCm38 mouse reference genome with default parameters. The mapped sequencing reads were assigned to Ensembl gene annotation with featureCounts function of Rsubread package (version 2.0.1) with parameters specific to paired end reads. Gene expression was quantified by the reads that fall into exon and normalized to FPKM (fragments per kilobase of exon model per million mapped reads) by edgeR (version 3.28.1). Differential expression analysis was also executed by edgeR. Specifically, the counts were normalized using TMM (trimmed mean of M-values) normalization method, then the common dispersion and tagwise dispersions were estimated, and genewise quasi-likelihood F-tests using GLMs in edgeR were performed to calculate p-values for fold changes.

#### 16S rRNA gene sequencing

To harvest viable mucosal-associated and luminal microbial communities from the cecum of indicated mice, animals were sacrificed and abdomen was opened under sterile conditions. The cecum was opened and manually extruded along the cephalocaudal axis to collect the fecal material into a clean tube for 16S rRNA extraction. 16S rRNA gene sequencing methods were adapted from the methods developed for the Earth Microbiome Project (Global patterns of 16S rRNA diversity at a depth of millions of sequences per sample; Ultrahigh-throughput microbial community analysis on the Illumina HiSeq and MiSeq platforms). Briefly, bacterial genomic DNA was extracted using DNA Isolation Kit (TIANGEN, DP328-02). The 16S rDNA V3-V4 region was amplified by PCR and sequenced in the MiSeq platform (Illumina) using the 2x250 bp paired-end protocol yielding pair-end reads. The primers used fornamplification (515F-806R) contain adapters for MiSeq sequencing and single-end barcodes allowing pooling and direct sequencing of PCR products on the Illumina MiSeq platform.^42^

#### 16S rRNA gene compositional analysis

Adapters and primers were removed from the raw sequencing data using trimmoatic to get clean data. The paired-end sequence reads were imported into a QIIME2 (version 2020.6) artifact, and then length trimming, denoising, chimera and PhiX removal were implemented with dada2 plugin with default parameters. The resulting feature table were summarized for further exploration and the resulting feature sequences were used to generate a tree using q2-phylogeny plugin for phylogenetic diversity analyses. The alpha diversity analyses were performed using q2-diversity plugin to compute several alpha and beta diversity metrics, and generate principal coordinates analysis (PCoA) plots using Emperor for each of the beta diversity metrics. Finally, Naïve Bayes classifier using Silva v132 reference taxonomy files were trained to classify the feature sequences using q2-feature-classifier plugin.

#### CDR3 sequencing

RNA was extracted from old pituitary the same as described in RNA-sequencing. cDNA synthesis was performed with Rever Tra Ace (TOYOBO, TRT-101) based on a mix of three gene-specific primers for the IgA locus (5’-ATCAGGCAGCCGATTATCAC-3’, 5’-TCTCCTTCTGGGCACTCG-3’, and 5’-TGAATGATGCGCCACTGT-3’). Then PCR with a primer binding in the constant Cα region 5’-GAGCTCGTGGGAGTGTCAGTG-3’ in combination with a promiscuous VH primer 5’-AGGTGCAGCTGCAGGAGTCTGG-3’ (binding to all VH genes) was performed to generate template libraries of rearranged IgA sequences. PCR conditions were as follows: 95°C, 4 min; 30× (94°C, 30 s; 62°C, 30 s; 72°C, 35 s); and 72°C, 10 min. Amplicons were purified by gel extraction (QIAquick Gel Extraction kit; QIAGEN) and quantified by NanoDrop™ 2000/2000c Spectrophotometers (Thermo Fisher Scientific). Then the amplicons were sent to deep sequencing.

#### CDR3 Sequence analysis

Sequences were assigned to individual samples according to their MID. Sequences lacking one or both primer sequences or shorter than 320 bp were excluded. Sequences were further analyzed with MiXCR (version 3.0.13), a universal tool for fast and accurate analysis of T- and B- cell receptor repertoire sequencing data. The whole analysis was performed using analyze amplicon command. Specifically, paired-end reads were merged and aligned to reference V/D/J and C genes, then clone-types were built and assembled from the alignments and exported to a human readable text file. The top 50 CDR3 were selected for generating the pie chart according to their percentages.

#### BCR amplification using nested PCR

RNA was extracted from young or old pituitary the same as described in RNA-sequencing. cDNA synthesis was performed with Rever Tra Ace (TOYOBO, TRT-101) based on a mix of three gene-specific primers for the IgA locus (5’-ATCAGGCAGCCGATTATCAC-3’, 5’-TCTCCTTCTGGGCACTCG-3’, and 5’-TGAATGATGCGCCACTGT-3’). Then cDNA was used as template for PCR with primers listed in Extended Data Table S2.

#### *In situ* RNA hybridization (RNAscope)

Pituitary was isolated from perfused mouse and fixed with 4% Paraformaldehyde (PFA) (Solarbio, P1110) overnight at room temperature. Paraffin embedded tissues were sectioned at 5μm and processed to RNAscope. RNAscope was performed using the Multiplex Fluorescence v.2 kit (Advanced Cell Diagnostics, 323100) according to the manufacturer’s protocol. Probes for mouse *Igkc* (414291) and *Igha* (414281-C2) were commercially available from the manufacturer, and secondary Opal 520 reagents (FP1487001KT, Akoya Biosciences) were diluted 1:500 in TSA buffer.

#### Quantitative RT-PCR

RNA was extracted from pituitary of indicated mouse as described in RNA-sequencing. Other tissues were prepared to homogenate in lysis buffer and RNAs were extracted with RNAprep pure Cell/Bacteria Kit. Concentration of RNA was measured and cDNA was synthesized using PrimeScript 1st strand cDNA Synthesis Kit (TaKaRa, 6110A) according to manufacturer’s instruction. Quantitative RT-PCR (qRT-PCR) was performed with the synthesized cDNA using TransStart Top Green qPCR SuperMix (TransGen Biotech, AQ131-04) in the CFX Opus 96 Real-Time PCR System (Bio-Rad) following manufacturer’s instruction. The primers sequences used for Igkc and Igha are Igkc-F: 5’-GTTGACCAAGGACGAGTATGAA-3’; Igkc-R: 5’-TTGAAGCTCTTGACAATGGG-3’; Igha-F: 5’- CCAGAAGGAGAATCCGTGAAA-3’; Igha-R: 5’-AGGAGGACAAGGAGGACAA-3’.

#### Western blotting

Deep-anesthetized mice were perfused via the aorta with cold PBS and dissected pituitary was lysate in TNE lysis buffer (50 mM Tris-HCl, pH 7.4, 150 mM NaCl, 0.5% NP40, 2 mM EDTA, Roche complete protease inhibitor set). The pituitary was triturated by pipetting and incubated on ice for 15 min, and centrifuged at 2,000g for 10 min. The supernatants were collected and 4X SDS loading buffer was added. The samples were heated at 98°C for 10 min and ready for western blotting. The antibodies used for western blotting were: Goat Anti-Mouse IgA alpha chain (HRP) (abcam, ab97235, 1:1000), Goat Anti-Mouse Kappa-HRP (Southernbiotech, 1050-05, 1:2000) and Anti-GAPDH mAb-HRP-DirecT (MBL, M171-7, 1:20000).

#### Immunofluorescence staining

For pituitary IgA staining, paraffin sections (5μm) were prepared as described in *in situ* RNA hybridization. The sections were de-paraffinized in isopropanol and graded alcohols, followed by antigen retrieval and blocked with SuperBlock™ (PBS) Blocking Buffer (Thermo Fisher Scientific, 37515) containing 0.2% Triton X-100 for 30 min and incubated with goat anti-mouse IgA-PE (SouthernBiotech, 1040-09, 1:200) antibody overnight at 4°C. After washing with PBST 3 times, sections were mounted with DAPI Fluoromount-G (SouthernBiotech, 0100-20). Section samples were imaged on a Nikon A1-R confocal microscope. For the IgA+ cell quantitation, each pituitary was taken at least 3 pictures and the IgA+ cells in each picture were quantitated and the average number was used as IgA+ cells per view of each mouse.

For intestinal and pituitary section staining, tissues from *Igha-cre; Ai6* or *Pou1f1-cre; Ai6* mice were fixed in 4% PFA in PBS at room temperature for one hour and embedded in OCT compound, followed by frozen and cryosection (20μm). Cryosections were fixed for 10 min in 4% PFA in PBS at room temperature and mounted with DAPI Fluoromount-G after washing or staining with goat anti-mouse IgA-PE. Images were taken on Olympus VS120.

For AAV-EGFP infected pituitary analysis, fixed pituitary was embedded in OCT compound, frozen, cryosectioned (20μm) and fixed for 10 min in 4% PFA in PBS at room temperature. The OCT was washed out in PBS and sections were mounted with DAPI Fluoromount-G. Samples were imaged on a Nikon A1-R confocal microscope.

#### FACS

For Epcam positive and ZsGreen positive cells analysis and isolation, single cell suspension preparation was described in pituitary gland dispersion. The cells were stained with indicated antibody together with DAPI (1μg/mL) and analyzed or sorted by gating DAPI negative and FITC positive population.

For EGFP positive cells quantitation in AAV infected mice, pituitary single cell suspension was prepared and stained with DAPI before analysis.

For IgA+ cell quantitation, pituitary was dissociated and single cell suspension was prepared firstly. Then the cells were fixed for 30 min in 4% PFA in PBS at room temperature. After washing with PBS twice, SuperBlock™ (PBS) Blocking Buffer containing 0.2% Triton X-100 was added for permeabilization and blocking at room temperature. One hour later, the cells were incubated overnight with goat anti-mouse IgA-PE (1:200) at 4°C. After washing with PBST 3 times, cells were centrifuged and resuspended in 250μL PBS and passed through 40μM cell strainer (Corning, 352340). During the staining, cell suspension was centrifuged at 5000rpm for 3min to remove supernatant each time before changing incubation buffer.

For CD19 staining of spleen and pituitary cells from *Igha-cre; Ai6* mouse, spleen live cell suspension was obtained by directly grinding the spleen and pituitary cell suspension was prepared as described in pituitary gland dispersion. Cells were passed through 40μM cell strainer and stained with APC anti-mouse CD19 Antibody (BioLegend, 115512, 1:500) and DAPI (1μg/mL).

Cell analysis and isolation were performed on a BD FACSAria sorter equipped with FACSDiva software (BD Bioscience). Data were analyzed with FACSDiva software.

#### Antibiotics treatment

IgA-high mice or *Igha-cre; Ai6* mice were divided into two groups (control and treatment) randomly. Antibiotics cocktail containing ampicillin (Sigma, A9393, 5g/L), vancomycin (Sigma, V2002, 2.5g/L), metronidazole (Sigma, M1547, 5g/L), neomycin (Sigma, N6386, 5g/L) and Amphotericin B (Sigma, A4888, 50mg/L) were used for the treatment group. The treatment group mice were firstly daily oral gavage with 500uL antibiotics cocktail for two weeks when they were one month old, and then oral gavage with 500uL antibiotics once every three days until die. 0.01% hydrogen chloride (HCl) was added to the drinking water of treatment group after the first two-week antibiotics gavage. Then the mice were sacrificed at 3-4 months old for pituitary analysis.

#### Microbiota transfer

Mice were sacrificed and abdomen was opened under sterile conditions. The cecum was opened and manually extruded along the cephalocaudal axis to collect the fecal material into a clean tube. Collected material was weighed and then diluted at a 1:20 ratio (g collected material: ml cryoprotectant). The resulting suspension was mashed through a 40μM cell strainer (Corning, 352350) to remove insoluble particles. Bacteria solution from indicated mice were transferred by three consecutive daily oral gavage procedures into Germ-Free (GF) mice. GF mice were housed and microbiota transfer were conducted in the isolator under gnotobiotic conditions in Institute of Laboratory Animals Science.

#### Bone marrow transplant

2-3 months old IgA-high or *Igha-cre; Ai6* recipient mice received 11 Gy gamma ray (Gammacell 1000) were transferred with 5-10 million donor bone marrow cells by tail vein injection. The donor cells for IgA-high mice were from wildtype or *Igha KO* indicated in figure and the donor cells for *Igha-cre; Ai6* mice were from wildtype or *Igha-cre; Ai6* as indicated. Bone marrow cells were isolated from the femur of indicated mice. Red blood cells were removed by treating the cells with Red Blood Cell Lysis Buffer (Beyotime, C3702-120ml) for 10 min at 4°C. Four weeks after bone marrow transfer, recipient mice were sacrificed and the pituitaries and spleen were isolated for further analysis.

#### AAV-EGFP infection

Adeno-associated virus (AAV) expressing EGFP was packaged by Genetic Screening Center of NIBS. Each control or IgA-high mouse was infected with 1× 10^10^ viral genome (v.g.) AAV by tail vein injection. Two weeks later, the pituitary was separated and EGFP positive cells were analyzed.

### Quantification and statistical analysis

The data are presented based on at least 3 independent experiments as mean ± SD. Statistical significance was calculated by two-tailed Student’s t test or one way ANOVA- HolmŠídák post hoc for multiple comparison (Prism software; GraphPad). ∗p < 0.05; ∗∗p < 0.01; ∗∗∗p < 0.001; ns, not significant. All of the statistical details can be found in the figure legends.

## Data Availability

**Data.** Transcriptomic and Single-cell RNA-seq data have been deposited at GEO and are publicly available as of the date of publication. Accession numbers are listed in the key resources table. The published article includes all datasets generated or analyzed during this study. All data reported in this paper will be shared by the lead contact upon request. **Code.** This paper does not report original code. Any additional information required to reanalyze the data reported in this paper is available from the lead contact upon request.
