# Supplementary material for: Gut bacteria induce IgA expression in pituitary hormone-secreting cells during aging
Source: iScience. 2023 Aug 26;26(10):107747. doi: 10.1016/j.isci.2023.107747 (PMC10492204; doi:10.1016/j.isci.2023.107747)
Supplement: Document S1. Figures S1–S6 and Tables S1 and S2 [file mmc1.pdf]

iScience, Volume *26*

Supplemental information

Gut bacteria induce IgA expression in pituitary hormone-secreting cells during aging

Yehua Li, Jiawen Wang, Rui Wang, Ying Chang, and Xiaodong Wang

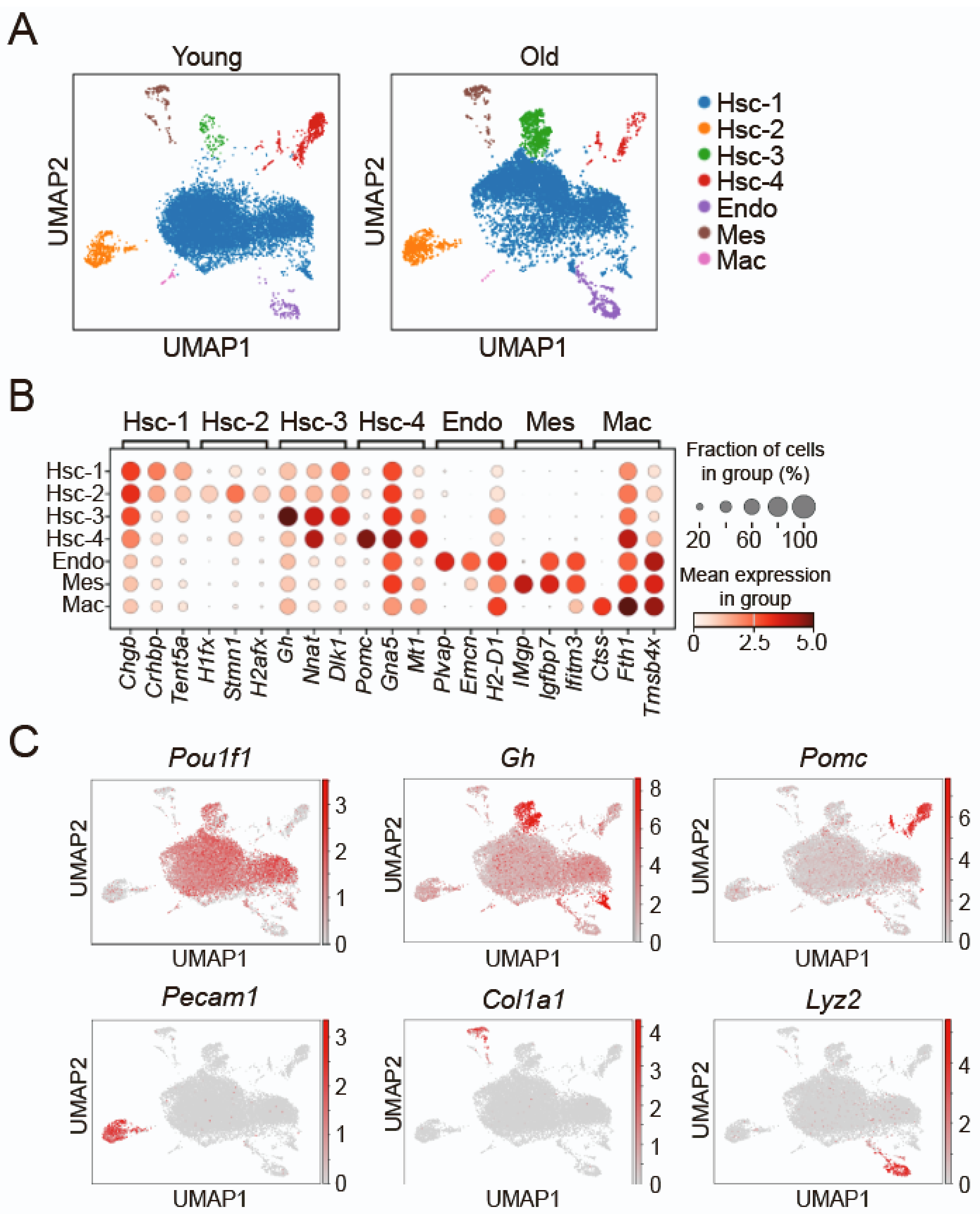

Figure S1. Single-cell RNA-seq (scRNA-seq) analysis of mouse pituitary during aging, related to Figure 1. (A) UMAP clustering of ScRNA-seq of the pituitary single-cell transcriptomes from young (2-3 months old) and old (18-24 months old) mice. (B) Dot plot showing the distribution of expression levels of different cell-type-enriched marker genes across 7 cell clusters. (C) Scatterplots showing the expression of *Pou1f1*, *Gh*, *Pomc*, *Pecam1*, *Col1a1* and *Lyz2* projecting on the UMAP plot. Gray to red indicate low to high expression levels.

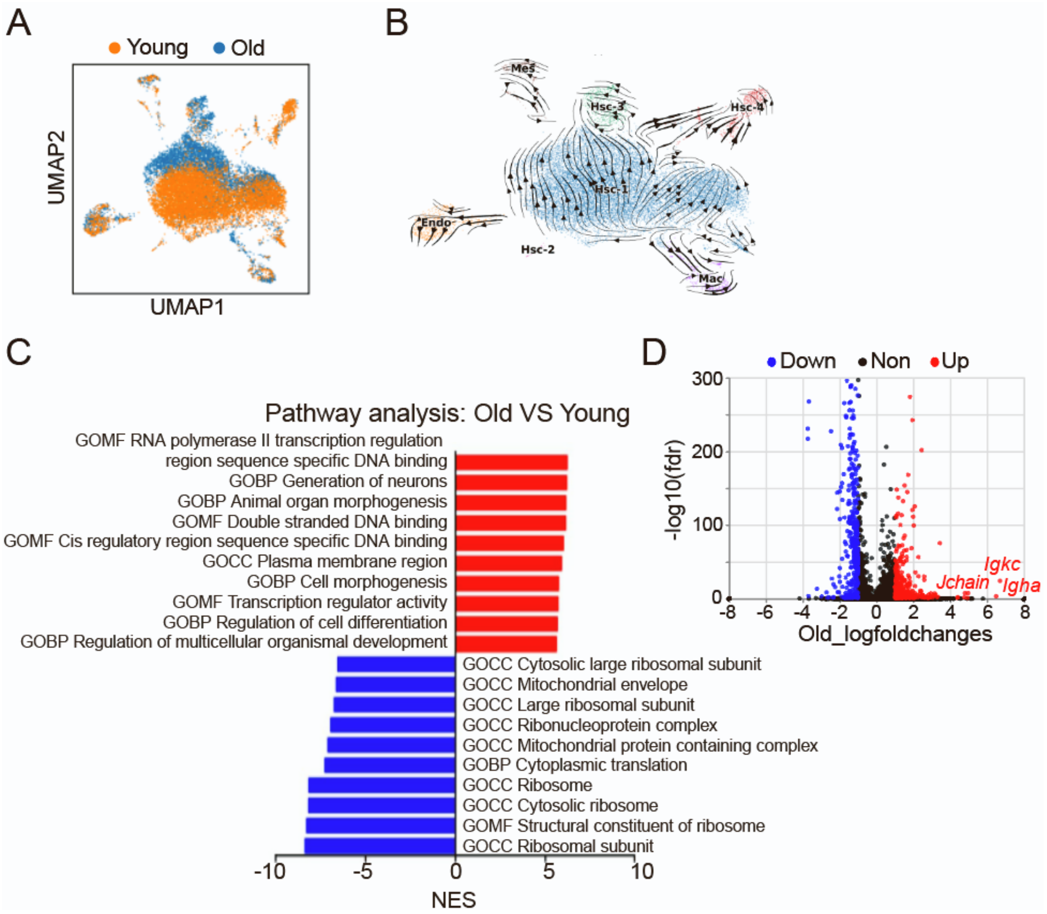

Figure S2. Gene expression change during pituitary aging, related to Figure 1. (A) UMAP clustering of pituitary cells colored by different ages. (B) RNA velocity analysis of young and old pituitary single cells. (C) The top 10 up- and down-regulated pathways in Hsc-1 population of old pituitary based on Gene set enrichment analysis (GSEA). Red and blue bars indicate pathways up- and down-regulated in old Hsc-1, respectively. NES, Normalized enrichment score. Gene Ontology (GO) dataset was selected for the analysis. GOMF, Gene Ontolgy Molecular Function; GOBP, Gene Ontology Biological Process; GOCC, Gene Ontology Cellular Component. (D) Volcano plots depicting the differentially expressed genes in Hormone-secreting cells-1 (Hsc-1) of young and old pituitaries. Blue dots indicate significantly down-regulated genes, red dots for significantly up-regulated genes, and black dots for unchanged genes.

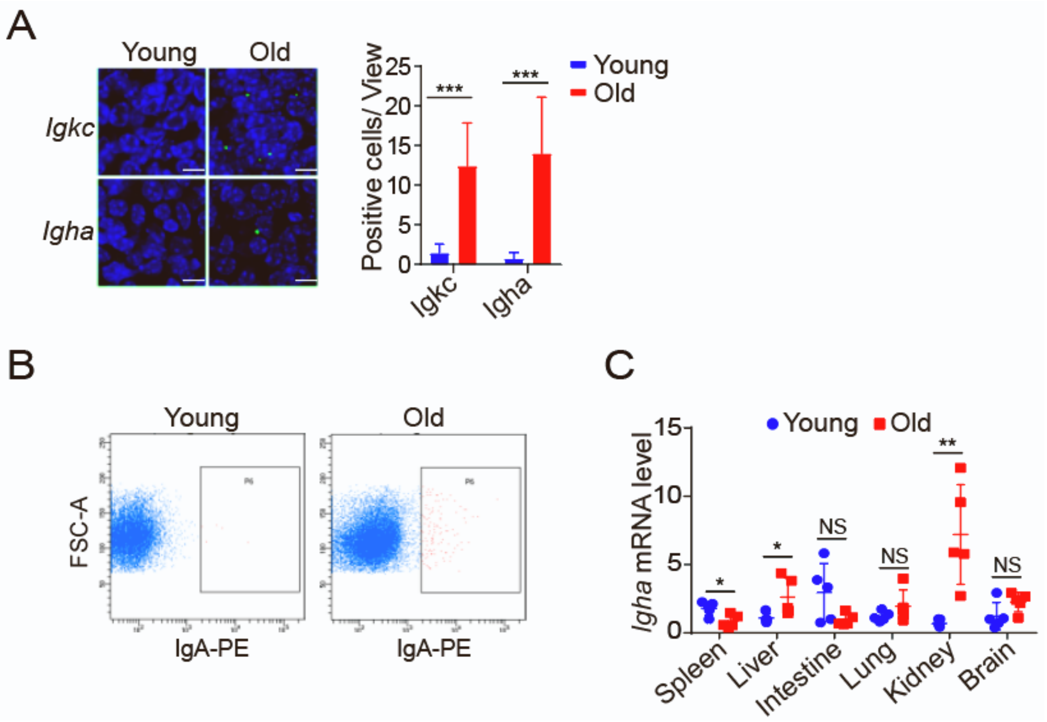

Figure S3. Polyclonal IgA increases in aged pituitary, related to Figure 2. (A) RNA *in situ* hybridization image (left) and quantification (right) of *Igkc* and *Igha* in young and old pituitary. Blue signal is DAPI and green signal is *Igkc* or *Igha* as indicated. scale bar 10μM. Images are representative results of three mice. (B) FACS data showing IgA+ cells in young and old pituitary. Pituitary cells are stained with IgA-PE antibody and analyzed by FACS as described in methods. Representative result is shown. (C) qRT-PCR result showing *Igha* mRNA level in different organs of young and old mice. n=5 mice per group. Data shown as mean ± s.d., unpaired two-tailed Student's t-test. *P<0.05, **P<0.01.

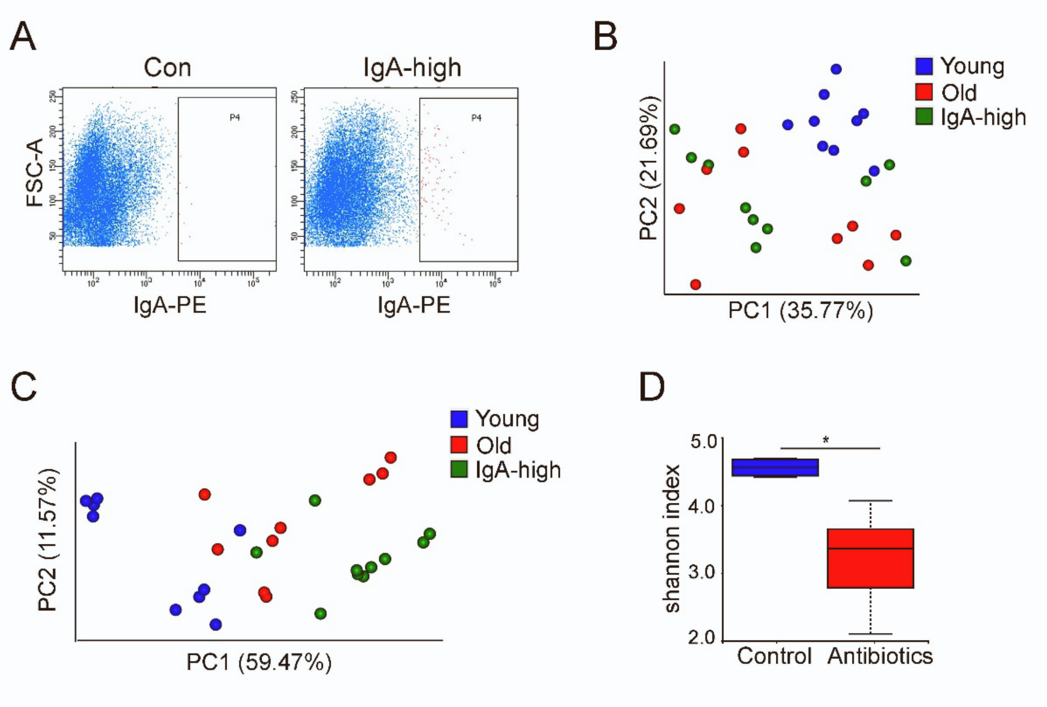

Figure S4. Gut microbiota diversity affects IgA expression in pituitary, related to Figure 3 and Figure 4. (A) Representative FACS data of IgA+ cells analysis in control and IgA-high mice. (B and C) 16S rRNA gene profiling data comparing the gut microbiome of young, old and IgA-high mice. Principal Coordinate Analysis (PCoA) of weighted UniFrac (B) and unweighted UniFrac (C) is shown. n=9 mice in young and old, n=10 mice in IgA-high. (D) Comparison of microbial diversity between control (n=4) and antibiotics (n=5) treated IgA high expression mice using Shannon index. Data shown as mean ± s.d., Kruskal Wallis test, $^{*}P<0.05$.

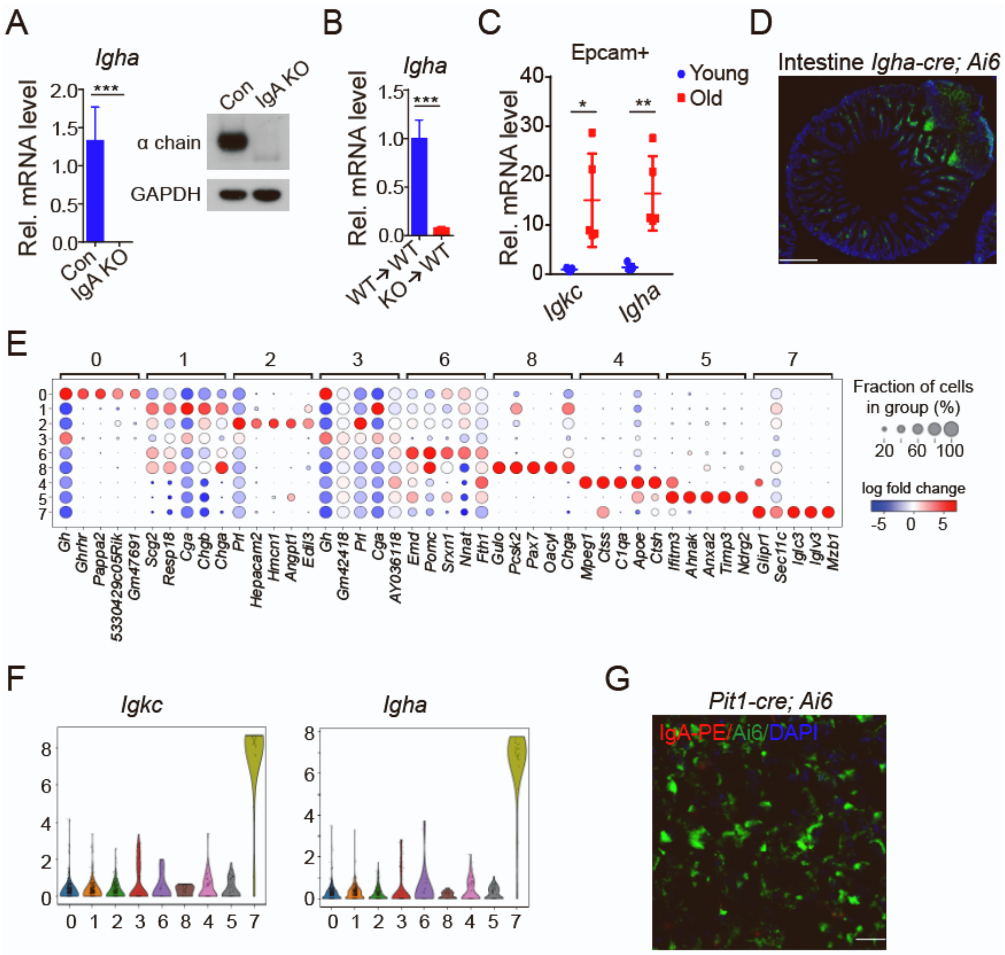

Figure S5. ScRNA-seq of ZsGreen positive pituitary cells from *Igha-Cre; Ai6* mice, related to Figure 5. (A) qRT-PCR (left) and immunoblot (right) of *Igha* (α chain) expression in wildtype (Con) and IgA knockout (IgA KO) mice spleen. n=4 mice per group for qRT-PCR. Representative data of three independent experiments for immunoblotting. Data shown as mean ± s.d., unpaired two-tailed Student's t-test, ***P<0.001. (B) *Igha* mRNA level in spleen of IgA-high mice transferred with bone marrow from wildtype (WT) and IgA knockout (KO) mice. The mice were sacrificed 4 weeks after bone marrow transfer and pituitary mRNA was analyzed by qRT-PCR. Data shown as mean ± s.d., unpaired two-tailed Student's t-test, ***P<0.001. (C)Expression levels of *Igkc* and *Igha* mRNA in Epcam+ pituitary cells from young and old pituitary. Epcam+ pituitary cells were sorted by FACS and mRNA levels of Igkc and Igha were detected by qRT-PCR. n=5 mice per group. Data shown as mean ± s.d., unpaired two-tailed Student's t-test, *P<0.05, **P<0.01. (D) Immunohistochemistry image of intestine from *Igha-cre; Ai6* mouse. Green signal is ZsGreen and blue signal is DAPI. Scale bar, 500μM. (E) Dot plot showing the differential expression levels of different cell-type-enriched marker genes across 9 cell clusters in ZsGreen positive pituitary cells from *Igha-Cre; Ai6* mice. (F) Violin plot shows expression of *Igkc* and *Igha* as log-normalized counts in ZsGreen positive pituitary cells from *Igha-Cre; Ai6* mice. Each dot represents expression level in one single cell. (G) Immunohistochemistry staining of IgA in old *Pit1-cre; Ai6* mouse pituitary. Green signal is ZsGreen, blue signal is DAPI and red signal is IgA. Scale bar, 20μM.

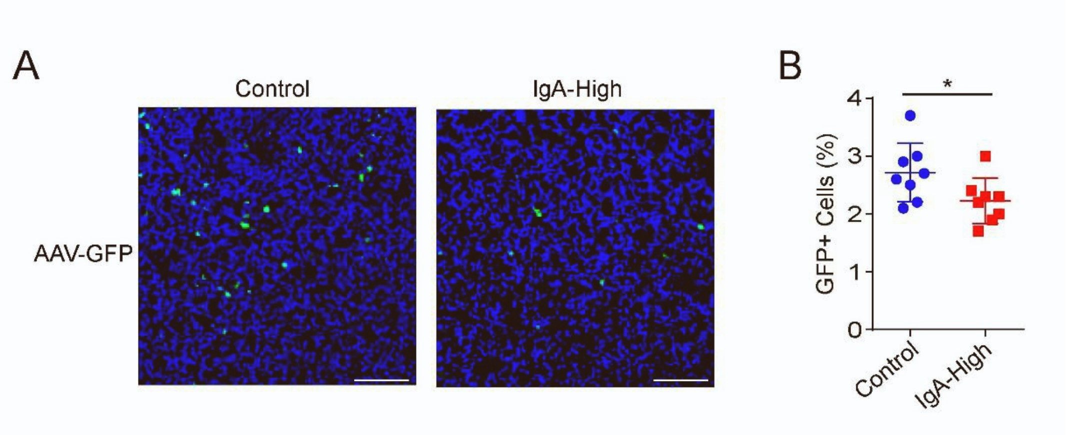

Figure S6. IgA in pituitary protects it from virus infection, related to Figure 5. (A) Image showing expression of EGFP in pituitary of control and IgA-high mice after Adeno-associated viruses (AAV) infection. Two weeks after AAV infection, mice are sacrificed and frozen sections of pituitary from indicated mice infected with AAV expressing EGFP are stained with DAPI. Green signal is EGFP and blue is DAPI. Scale bar, 100μM. (B) Quantitation of EGFP+ cells in AAV infected pituitary. Pituitary of control and IgA-high mice infected by AAV expressing EGFP are subjected to FACS, and the percentage of EGFP+ cells is quantitated. n=8 mice per group. Data shown as mean ± s.d., unpaired two-tailed Student’s t-test, *P<0.05.

Table S1. VH and VL expression change in aged pituitaries, related to Figure 2.

VH and VL segment list

V Chains	Young1	Young2	Young3	Young4	Old1	Old2	Old3	Old4
VH1-83	0	0	0	0	0.176973	0	0	0
VH1-82	0	0	0	0.141647	0	0	0	0
VH1-81	0	0	0	0	0.884864	0	0	0.162886
VH1-80	0	0	0	0	0	0.308227	0	0
VH1-78	0	0	0	0	0.165206	0.287733	0.276003	0
VH1-76	0	0	0	0	1.181582	0.587976	0	0
VH1-74	0	0	0	0	0	0.308227	0	0
VH1-72	0	0	0	0	0	0.1366	0.131031	0
VH1-71	0	0	0	0	0	0	0.144536	0
VH8-12	0	0	0	0	0.173512	0.1511	0.28988	0.159701
VH1-66	0	0	0	0	0	0.40672	0	0
VH8-11	0	0	0	0	0	0	0.28988	0
VH1-64	0	0.164146	0	0	0.153376	0	0.256239	0.423504
VH1-62-2	0	0	0	0	0.328664	0	0	0
VH1-62-1	0	0	0	0	0	0.154997	0	0
VH1-59	0	0	0	0	0	0.308227	0	0
VH1-58	0	0	0	0	0	0.154113	0.14783	0
VH8-8	0	0	0	0	1.561612	0.6044	0	0
Ighv8-7	0	0	0	0	0.20915	0	0	0
VH1-55	0	0	0	0	0	0	0.250065	0.275533
VH1-53	0	0	0	0	0	0.46234	0	0
VH1-52	0	0	0	0	0	0.308227	0	0.814431
VH1-50	0	0	0	0	0	0.138702	0	0
VH8-5	0	0	0	0	0	0.7555	0	0
VH1-43	0	0	0	0	0	0	0.14783	0
VH1-42	0	0	0	0	0	0.154113	0	0
VH1-39	0	0	0	0	0.176973	0.154113	0	0.325772
VH1-37	0	0	0	0	0	0.154113	0	0
VH1-33	0	0	0	0	0	0	0.148253	0
VH1-26	0	0	0.55782	0	0.530919	0.46234	0.886982	0.162886
VH1-22	0	0	0	0	0	0.46234	0	0.162886
VH1-20	0	0	0	0	0	0.154113	0	0
VH1-19	0	0	0	0	0.153376	0	0.256239	0
VH1-18	0	0	0	0	0.530919	1.541134	0	0.162886
VH1-15	0	0	0	0	0	0	0.295661	0
VH1-14	0	0	0	0	0	0	0	0.142222
VH1-12	0	0	0	0	0	0.280279	0	0
VH1-10	0	0	0	0	0.174979	0	0	0
VH1-9	0	0	0	0	0.176973	0.46234	0	0
VH15-2	0	0	0	0	0	0.45457	0	0
VH1-7	0	0	0	0	0	0	0.267466	0
VH10-3	0	0	0	0	1.48291	0.143485	0	0
VH1-5	0	0	0	0	0.314519	0	0	0
VH1-4	0	0	0	0	0.176973	0	0	0
VH10-1	0	0	0	0	0.346058	0.602716	0.578145	0.159256
VH6-3	0	0	0	0	0	0	0.145754	0
VH13-2	0	0.185179	0	0	0	0.150679	0.289072	0
VH3-6	0	0	0	0	0.532435	0.618215	0	0.163352
VH3-3	0	0	0	0	0	0	0.148253	0.163352
VH14-4	0	0	0	0	0	0	0	0.488659
VH7-3	0	0	0	0	0.172071	0	0	0
VH9-3	0	0	0	0	0.353946	0.616454	0.739152	0

VH14-3	0	0	0	0	0.467049	0	1.690602	0
VH11-2	0	0	0	0	0	0.704041	0.675338	0.868138
VH14-2	0	0	0	0	0.353946	1.078794	0	0
VH11-1	0	0	0	0	0	0.459721	0.146993	0
VH4-1	0	0	0	0	0	0.130661	0.125334	0
VH14-1	0	0	0.13945	0	0.176973	1.232907	0.295661	0
VH2-9	0	0	0	0	0	0	0.286677	0
VH5-17	0	0	0	0	0.153376	0.133565	0.256239	0.282336
VH5-16	0	0	0	0	0.163467	0.569408	0.409646	0
VH5-15	0	0	0	0	0	0	0.431206	0
VH2-9-1	0	0	0	0	0	0	0.140619	0.154941
VH2-5	0	0	0	0	0	0.112695	0	0
VH5-9	0	0	0	0	0	0.129411	0	0
VH2-4	0	0	0	0	0.177478	0	0	0
VH5-6	0	0	0	0	0.879851	0.30648	0.293986	0
VH2-3	0	0	0	0	0	0.31178	0.149534	0.164764
VH5-4	0	0	0	0	0	0	0	0.26905
VH2-2	0	0	0	0	0.52941	0	1.326694	0
V13	0	0	0	0	0.163038	0.567914	0	0
V12	0	0	0	0	0.50916	1.477973	0	0
V11	0	0	0.12519	0.279672	1.270946	0.830084	0.92895	0.731113
Vk1-135	0.1476271	0	0	0	0.315317	0.961057	0	0.725546
Vk1-133	0	0	0	0	0	1.590994	0	0.458607
Vk9-129	0	0	0	0	0	0.30648	0	0
Vk17-127	0	0	0	0	0	0.452037	0	0
Vk14-126	0	0	0	0	0	1.511	2.029158	0.479104
Vk9-124	0	0	0	0	0	0.30648	0.734964	0
Vk1-122	0	0	0	0	0.162187	0	0	0.149277
Vk17-121	0	0	0	0	0	0.149019	0	0
Vk9-120	0	0	0	0	0.703881	0.459721	0	0.48589
Vk1-117	0	0	0.12116	0	2.152586	2.945702	4.495288	2.122762
Vk2-116	0	0	0	0	0.171595	0	0	0
Vk2-112	0	0	0	0	0	0	1.724825	0
Vk14-111	0	0	0	0	0	0.881964	0	0.155362
Vk1-110	0	0.663133	0	0	3.253035	1.48387	1.68217	1.283186
Vk2-109	0	0	0	0	1.660895	0	0	0
Vk16-104	0	0	0	0	0.492996	0.429316	0	0
Vk15-103	0	0	0	0	0	0.43275	0.138369	0.609846
Vk14-100	0	0	0	0	0	0.459721	0	0.161963
Vk10-96	0	0	0	0	0.692117	0.150679	0.433608	0.637026
Vk10-95	0	0	0	0	0.179013	0	0	0
Vk10-94	0	0	0.70531	0	0.179013	0.31178	0.747672	0.164764
Vk4-90	0	0	0	0	0.17647	0.153676	0	0.162423
Vk12-89	0	0.393367	0	0	0	0.480122	0.460549	0
Vk4-86	0	0	0.12949	0	0	0	0	0
Vk13-84	0	0	0	0	0.358026	0	0.149534	0
Vk4-81	0	0	0	0	0.173512	0	0	0
Vk4-80	0	0	0	0	0.713994	0	0	0
Vk4-75	0	0.185179	0	0	0	0	0	0
Vk4-74	0.1476271	0	0.12423	0.138771	0.315317	0.411882	0.263393	0
Vk13-73-1	0	0	0	0	0	0.270469	0	0
Vk4-73	0	0	0	0	0	0.1511	0.14494	0
Vk4-72	0	0	0.12176	0	0.463563	0.538247	0	0
Vk4-71	0	0	0	0.154452	0	0.152807	0.146578	0

Vk4-70	0.3069396	0.701626	0.12915	0	0	0.999094	0.273818	0
Vk4-69	0.167141	0.764128	0.70328	0.314229	0.713994	0.155442	1.640153	0.16429
Vk4-68	0.1476271	0.168729	0	0	0	0.68647	0.131697	0.145109
Vk12-66	0.167141	0	0	0	0	0	0	0
Vk4-63	0	0.377722	0	0	0.17647	0.614702	0.442231	0.649694
Vk4-62	0	0.187794	0	0	0.526419	0	0.439733	0
Vk13-61-1	0	0	0	0	0	0	0.350598	0
Vk4-61	0	0.174029	0	0	0	0	0.543335	0
Vk4-60	0	0	0	0	0	0	0	0.159701
Vk4-59	0	0	0	0	0	0.293988	0.141001	0
Vk4-58	0	0	0	0	0.173512	0	2.174098	0
Vk13-57-2	0	0	0	0	0	0.160993	0	0
Vk4-57-1	0.1559385	0	0.13123	0	2.164952	0.145024	0.278222	0.459837
Vk4-57	0	0	0	0	0.52941	0	0	0
Vk13-56-1	0.5566034	0	0	0	0	0.258822	0	0
Vk4-56	0	0.380969	0	0.156664	0	0.154997	0	0
Vk13-55-1	0.1888476	0	0	0	0	0.175629	0.168469	0.371254
Vk4-55	0	0.350813	0	0	0	0.428183	0.136909	0
Vk4-54	0.1643081	0	0	0	0.526419	0.152807	0	0
Vk4-53	0	0	0	0	0	0.736973	0.989698	0
Vk4-50	0	0	0	0	0	0.153676	0	0
Vk5-48	0	0	0	0	1.20784	0	0.576539	0
Vk12-46	0	0	0	0	0	0.143485	0.27527	0
Vk12-44	0.14505	0	0	0	0.154906	0.674486	0.129398	0.285152
Vk5-43	0	0	0	0	0.491695	0.999094	0.547636	0
Vk12-41	0	0	0.14106	0.157567	0	0.62356	0	0.823819
Vk12-40	0.1676227	0.191582	0	0	0	0.15589	0	0
Vk5-39	0	0	0	0	0.537039	0	0	0.494292
Vk8-34	0	0	0	0	0	0.296404	0	0
Vk6-32	0	0	0	0	0	0.443392	0	0
Vk8-30	0.1580572	0	0	0	0	1.616934	1.269012	0.155362
Vk6-29	0	0	0	0	0	0.14425	0	0
Vk8-27	0	0	0	0	0	0.300521	0	0
Vk8-26	0	0	0	0	0	0.145805	0	0
Vk6-25	0	0	0	0	0.817335	1.138817	0	0.300911
Vk8-24	0	0	0	0	0	0.296404	0	0
Vk6-23	0	0	0.2653	0	0	0.293191	0.562477	0
Vk8-21	0	0	0.12814	0	0	0.141607	0	0.449003
Vk6-20	0	0	0	0	0.331293	1.154001	0	0
Vk8-19	0	0	0	0	0.167885	0.146199	0	0
Vk8-18	0	0	0	0	0.314519	0	0	0
Vk6-17	0	0	0	0	0.823839	0.430455	0.137635	0
Vk6-15	0	0	0.14106	0	0.716052	0.62356	2.392552	2.14193
Vk6-13	0	0	0	0	1.903321	1.054754	0.144536	0
Vk3-12	0	0	0	0	0.692117	0.602716	0	0
Vk3-7	0	0	0	0	0.173029	0.602716	0.722681	0
Vk3-5	0	0	0	0	0.162611	1.416068	0.679168	0
Vk3-4	0	0	0.13597	0	0	0.150261	0	4.28798
Vk3-2	0	0	0	0.144645	0.657328	1.574158	0.274542	1.05876

Table S2. Primers for BCR amplification, related to STAR Methods.

Ighv1-84-F	GGATGGAGCTGGATCTTTCTT
Ighv1-82-F	GGACGGATTTATCCTGGAGATG
Ighv1-81-F	GTATAAGCTGGGTGAAGCAGAG
Ighv1-80-F	CTACTGGATGAACTGGGTGAAG
Ighv1-14-F	GGCTTCTGGATACACATTCACTA
Ighv1-13-F	CAAAGGCAAGGCCACATTTAC
Ighv1-11-F	TGAGGACCCTGCTGTCTATTA
Ighv1-9-F	ACTGAGGACTCTGCCATCTAT
Ighv1-5-F	GACAGGGTCTGGAATGGATAG
Ighv1-4-F	GGCTTCTGGCTACACCTTTA
Igha-R	TCAGGATTTCTCAGGCCATTC
